# The Influence of Heat Treatment on the Photoactivity of Amine-Modified Titanium Dioxide in the Reduction of Carbon Dioxide

**DOI:** 10.3390/molecules29184348

**Published:** 2024-09-13

**Authors:** Iwona Pełech, Piotr Staciwa, Daniel Sibera, Konrad Sebastian Sobczuk, Wiktoria Majewska, Ewelina Kusiak-Nejman, Antoni W. Morawski, Kaiying Wang, Urszula Narkiewicz

**Affiliations:** 1 Department of Inorganic Chemical Technology and Environment Engineering, Faculty of Chemical Technology and Engineering, West Pomeranian University of Technology in Szczecin, Pułaskiego 10, 70-322 Szczecin, Poland; piotr.staciwa@zut.edu.pl (P.S.); daniel.sibera@zut.edu.pl (D.S.); sk43128@zut.edu.pl (K.S.S.); ewelina.kusiak@zut.edu.pl (E.K.-N.); antoni.morawski@zut.edu.pl (A.W.M.); urszula.narkiewicz@zut.edu.pl (U.N.); 2 Department of Construction and Road Engineering, Faculty of Civil and Environmental Engineering, West Pomeranian University of Technology in Szczecin, Piastów 50a, 70-311 Szczecin, Poland; 3 Department of Microsystems, University of South-Eastern Norway, 3184 Horten, Norway; kaiying.wang@usn.no

**Keywords:** titanium dioxide, carbon dioxide, photocatalyst, amines, hydrogen evolution

## Abstract

Modification of titanium dioxide using ethylenediamine (EDA), diethylamine (DEA), and triethylamine (TEA) has been studied. As the reference material, titanium dioxide prepared by the sol–gel method using titanium(IV) isopropoxide as a precursor was applied. The preparation procedure involved heat treatment in the microwave reactor or in the high-temperature furnace. The obtained samples have been characterized in detail. The phase composition was determined through the X-ray diffraction method, and the average crystallite size was calculated based on it. Values for specific surface areas and the total pore volumes were calculated based on the isotherms obtained through the low-temperature nitrogen adsorption method. The bang gap energy was estimated based on Tauc’s plots. The influence of the type and content of amine, as well as heat treatment on the photocatalytic activity of modified titanium dioxide in the photocatalytic reduction of carbon dioxide, was determined and discussed. It was clear that, regardless of the amount and content of amine introduced, the higher photoactivity characterized the samples prepared in the microwave reactor. The highest amounts of hydrogen, carbon monoxide, and methane have been achieved using triethylamine-modified titanium dioxide.

## 1. Introduction

Air pollution is one of the most serious adverse effects of economic growth that affects everyday living. Burning fossil fuels leads to uncontrolled increases in greenhouse gas concentrations in the atmosphere. As a result, progressive global warming puts the global ecosystem out of order. Carbon dioxide emission, due to the tremendous amounts of this gas produced every year, is perceived to be one of the biggest concerns given climate change. Worldwide emission of this gas is constantly growing, i.e., in 2023, it reached 35.8 Gt [1], so the containment of CO_2_ emissions for years to come will be a difficult task.

Taking this into consideration, the scientific community struggles to find means to counter this effect. In order to mitigate the CO_2_ concentration in the atmosphere, several solutions have been proposed. Among others, carbon capture and storage [2], electrochemical conversion [3], catalytic conversion [4] and photocatalytic reduction [5] can be distinguished. The latter solution is especially attractive due to the possibility of the use of solar energy and the possibility of acquiring useful products from CO_2_. 

Photocatalytic CO_2_ reduction is a green, photo-induced process. Through the application of irradiation, carbon dioxide molecules can be transferred into useful products such as carbon monoxide, methane or hydrogen. An important component of photocatalytic CO_2_ reduction is a proper photocatalyst. In general, three steps can be distinguished in a photocatalytic process: (I) the generation of photogenerated carriers, (II) transferring the generated electrons and holes to the surface of the photocatalyst, (III) the catalytic reaction on the surface of the photocatalyst [6].

Research regarding the search for a proper semiconductor in order to enhance the effectiveness of photocatalytic CO_2_ reductions is in the pipeline. A suitable material must meet several requirements, i.e., a relatively narrow bandgap and a low recombination rate of charges strongly affect the photocatalysis efficiency. Finally, the photocatalyst should be cost-effective and stable during reaction [7]. There are several semiconductors that can be stated as promising photocatalysts, i.e., ZnO [8], Fe_2_O_3_ [9], Cu_2_O [10] or TiO_2_ [11]. However, none of the existing semiconductors meet the requirements for large-scale CCU (carbon capture and utilization) application, and further improvement of the efficiency of these materials is strongly advised. 

Among existing photocatalysts, titanium dioxide is a very promising material because of its low cost, non-toxicity and availability. Moreover, many publications attest to the ease of its modification. It was proven that, through modification with Ag [12], Cu [13], I [14], Co [15], and N [16], titanium dioxide can become a highly efficient CO_2_-reducing photocatalyst. 

In the available papers, it is reported that amines, as titania modifiers, increase their adsorptive properties towards CO_2_. Chen et al. [17] reported the increase in photocatalytic reduction of CO_2_ using amine-modified brookite TiO_2_ nanorods coupled with CuxS. Song et al. [18] prepared TiO_2_ nanotubes modified by three kinds of amines (ethylenediamine, polyetherimide and tetraethylenepentamine). TEPA-modified TiO_2_ nanotubes showed the highest CO_2_ adsorption capacity. Ota et al. [19] developed a synthesis method of new amorphous titanium dioxide nanoparticles with a diameter of 3 nm, a high surface area and a large amount of OH groups. Amorphous TiO_2_ nanoparticles were successfully modified with ethylenediamine and showed a higher CO_2_ adsorption capacity than conventional TiO_2_ and mesoporous SiO_2_. Jiang et al. [20] developed a new low-cost, highly selective and stable sorbent-based pre-combustion CO_2_ capture. Experimental results have shown that the selectivity of TiO_2_ for separation of CO_2_ from CO_2_/CH_4_ mixture can be significantly improved via amine modification. Ma et al. [21] modified TiO_2_ using different amines, including diethylenetriamine (DETA), triethylenetetramine (TETA), and tetraethylenepentamine (TEPA), for CO_2_ capture. Experimental results revealed that CO_2_ uptake capacities of the titania composite sorbents increase with amine loading but decrease with the size of impregnated amines. The same conclusion, that the amine-modified materials exhibited enhanced CO_2_ uptake compared to the initial titania, have been presented in our previous works [22,23].

As shown above, the amine modification of the photocatalyst has a positive effect on the enhancement of CO_2_ adsorption. The influence of this type of modification on the photocatalytic activity of TiO_2_ was reported. Mendonça et al. [24] used the activated carbon modified with ethylenediamine and impregnated with TiO_2_ for improved photodegradation of sulfamethazine. Bao et al. [25] synthesized photocatalytically active N-doped TiO_2_ nanoparticles by a one-pot hydrothermal method without calcination using structurally different amine sources as dopants under soft-chemistry conditions. TiO_2_ modified with diethylamine was a suitable candidate due to its high visible-light absorption ability and having the highest efficiency in regard to the photodegradation of reactive brilliant red dye. Liao et al. [26] stated that amine functionalization of TiO_2_ nanoparticles substantially increases the affinity of CO_2_ on TiO_2_ surfaces for more effective CO_2_ activation. It also greatly enhances the photocatalytic rate of CO_2_ reduction into CH_4_ and CO. Karawek et al. [27] prepared a sandwich-type composite consisting of two 2-dimensional nanostructures (2D−2D) using the heterostructure of titanium dioxide nanosheets (TNS) and graphene oxide (GO). Alkanolamine MEA, DEA, and TEA were tested to promote the photoactivity of TNS in photoreducing CO_2_. The TEA−TNS performed better than DEA−TNS and MEA−TNS due to increased CO_2_ loading and faster CO_2_ desorption rates. The best results came from the TEA-[Cu-TNS/GO] composite, in which the TEA was grafted onto the Cu-TNS/GO, attracting CO_2_ for the photoreduction, and the copper ions enhanced the charge separation characteristics of the TNS. Jin et al. [28] demonstrated that surface modifications with primary amines on TiO_2_ increase the activity in Co or methane production, regardless of metalcocatalysts. 

In the present work, amines with different boiling points, and featured different numbers of nitrogen and carbon atoms were used to modify TiO_2_: ethylenediamine (EDA, C_2_H_8_N_2_, 116.9 °C), diethylamine (DEA, C_4_H_11_N, 55.5 °C), and triethylamine (TEA, C_6_H_15_N, 89.8 °C). Two different methods were used for modification—a microwave oven operating under pressure and a thermal oven for high-temperature processing—to determine how these conditions affect the photoactivity of the samples.

## 2. Results and Discussion

**Figure 1:**
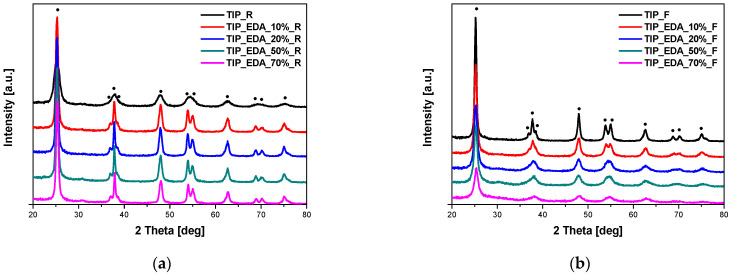
X-ray diffraction patterns of EDA-modified titania samples heated in the microwave reactor (**a**) and high-temperature furnace (**b**). Reflexes attributed to anatase are marked as •.

**Figure 2:**
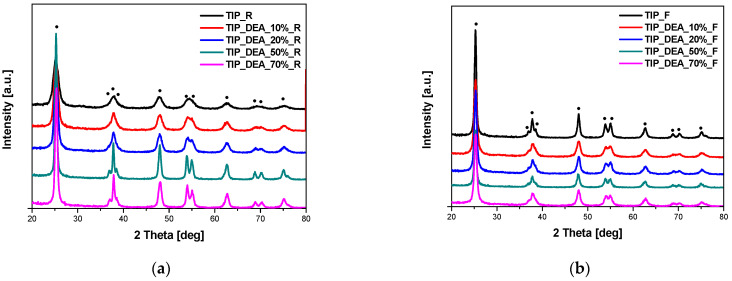
X-ray diffraction patterns of DEA-modified titania samples heated in the microwave reactor (**a**) and high-temperature furnace (**b**). Reflexes attributed to anatase are marked as •.

**Figure 3:**
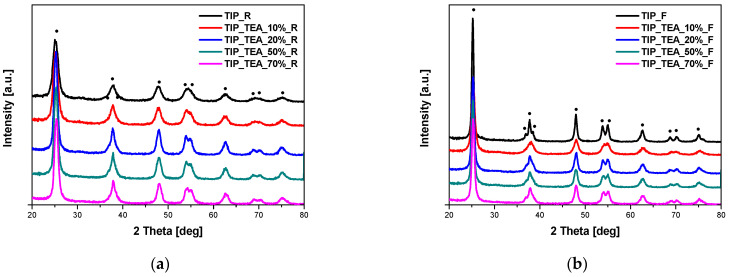
X-ray diffraction patterns of TEA-modified titania samples heated in the microwave reactor (**a**) and high-temperature furnace (**b**). Reflexes attributed to anatase are marked as •.

The phase composition of the obtained materials was studied using the X-ray diffraction method. In Figure 1, the diffraction patterns of the EDA-modified titania heated in the microwave reactor (Figure 1a) and high-temperature furnace (Figure 1b) are presented. All the visible reflexes were assigned to the anatase phase (ICDD 01-073-1764), and no other phases were identified in the samples. Exactly the same results were obtained for DEA-modified titania (Figure 2) and TEA-modified titania (Figure 3). 

Based on the reflex located at the 2 theta angle of approximately 25° and assigned to the anatase phase, the average crystallite size was calculated. The obtained values are presented in Table 1. It was found that the average crystallite size for the reference sample obtained in the microwave reactor (TTIP_R) was smaller (14.3 nm) than for the same material obtained in the high-temperature furnace (TTIP_F, 21.9 nm); this was probably related to the difference in heating temperatures (400 °C in the tubular furnace and 250 °C in the microwave reactor). Microwave heating is an in situ energy conversion mode and radically differs from traditional heating processes. According to the available literature [29,30,31,32], microwave heating has significant advantages over conventional heating procedures and can heat materials to high temperatures with ultra-fast heating rates in a short time with high energy efficiency. Using this method, low values of the anatase average crystallite size can be achieved, as was mentioned above and in [33,34]. Higher temperatures in the case of traditional heat treatment result in the formation of larger particles [35,36].

The materials modified with DEA and TEA and prepared in the microwave reactor had lower values of average crystallite size than the appropriate reference sample. For both DEA- and TEA-modified titania, the average crystallite size was very similar, and no significant differences were noted. All the calculated values ranged from ~7 nm to ~10 nm. In the case of ethylenediamine, the amine content does not have a significant impact on the average crystallite size as well, which ranged from 15.4 nm for TTIP_EDA_10%_R to 16.9 nm for TTIP_EDA_70%_R. Higher values of the average crystallite size were achieved for the materials modified with EDA in comparison with TTIP_R, and especially with the samples modified using DEA and TEA. A similar dependence was noticed at [37], where the authors calculated the crystallite size by the Sherrer’s formula and found that the crystallite sizes of the deposits in the case of 3-methoxypropylamine and dimethylamine, compared to ethanolamine, were approximately 17% and 24% less, respectively. Sun et al. [38] stated that, in low-pH conditions, the quantity of hydroxyl groups in hydrolysis is small, which prevents the crystallization of the samples and the growth of the TiO_2_ crystallites. With increasing pH, more hydroxyl groups are available for the hydrolysis, which leads to better crystallization of TiO_2_ and greater crystallite sizes [39,40]. Considering that EDA is the most basic amine among those used in studies, the above explanation may indicate why we obtained smaller crystallites of anatase using diethylamine and triethylamine.

All samples heated in the high-temperature furnace, regardless of the type of amine used for modification, were characterized by a lower average crystallite size than the reference sample, TTIP_F. For titania modified with EDA and heated in the tubular furnace, a slight decrease in the average crystallite size was noticed together with an increase in amine content in the sample. And so, for TTIP_EDA_10%_F, the average anatase crystallite size equalled 13.8 nm, whereas, for TTIP_EDA_70%_F, 9.0 nm was calculated. Similar to materials heated in the microwave reactor, the average crystallite size obtained for both DEA- and TEA-modified titanium dioxide, was not significantly different. However, it should be noted that these differences, although small, were greater than in the case of samples heated in the microwave reactor. The values ranged from 12.7 nm (TTIP_DEA_10%_F) to 17.0 nm (TTIP_DEA_50%_F) and from 10.3 nm (TTIP_TEA_10%_F) to 14.5 nm (TTIP_TEA_20%_F). 

**Figure 4:**
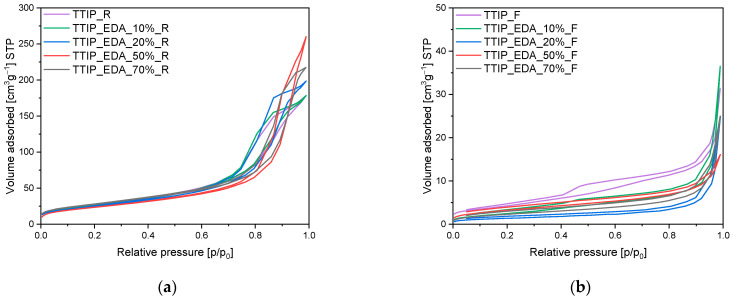
N_2_ sorption isotherms of the EDA-modified titania dioxide prepared using (**a**) a microwave reactor and (**b**) a high-temperature furnace.

**Figure 5:**
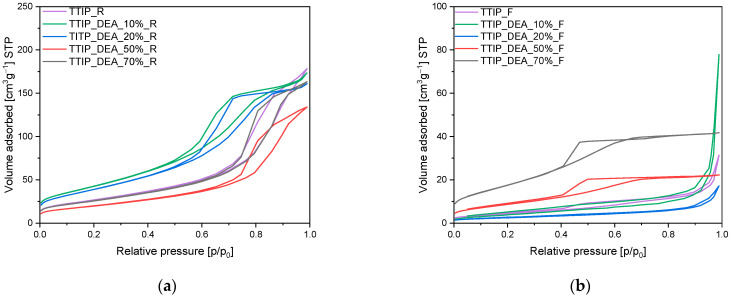
N_2_ sorption isotherms of the diethylamine-modified titanium dioxide prepared using (**a**) microwave reactor and (**b**) high-temperature furnace.

**Figure 6:**
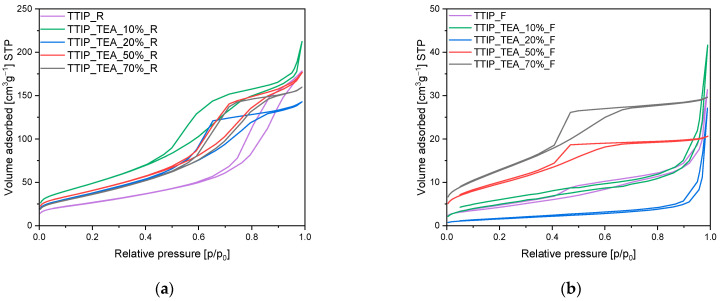
N_2_ sorption isotherms of the triethylamine-modified titanium dioxide prepared using (**a**) microwave reactor and (**b**) high-temperature furnace.

The obtained samples were also characterized using the low-temperature nitrogen adsorption method, and, based on the adsorption isotherms presented in Figure 4, Figure 5 and Figure 6, different parameters have been calculated. The values of specific surface areas, the total pore volumes and the volumes of pores are presented in Table 1. 

In Figure 4, the adsorption isotherms for EDA-modified titania prepared using a microwave reactor (Figure 5a) and high-temperature furnace (Figure 4b) are presented. For both the reference materials TTIP_R (Figure 4a) and TTIP_F (Figure 4b), the adsorption isotherms are of type II according to the IUPAC system, and their shape is characteristic of macroporous materials. For these samples, a H3 type of hysteresis loop is also observed [41,42], starting at a relative pressure of 0.45 p/p_0_ and ending at a relative pressure of approximately 1 p/p_0_, which indicates that the discussed materials are dominated by macropores not completely filled with pore condensate [41,42]. For the samples modified with EDA, regardless of the type of heat treatment, type II isotherms were obtained according to the IUPAC system, which is characteristic of macroporous materials. For the EDA-modified titania prepared using a microwave reactor, hysteresis loops of the H3 type were noticed, suggesting the presence of a pore network consisting of macropores [41,42]. On the contrary, in the case of EDA-modified titania prepared using a high-temperature furnace, hysteresis loops of the H4 type have been visible [41,42], and their presence indicates the existence of pores with the shape of narrow slits [41,42].

In Figure 5, the adsorption isotherms for diethylamine-modified titanium dioxide prepared using a microwave reactor (Figure 5a) and high-temperature furnace (Figure 5b) are shown. For DEA-modified titanium dioxide heated in the microwave reactor, similarly to the case of the reference material TTIP_R, the isotherms of type II were also obtained and the hysteresis loops of the H3 type were observed. For materials modified with an amine content of 10 wt.% and 20 wt.%, the hysteresis loop starts at a relative pressure of 0.5 p/p_0_ and ends at a relative pressure of approximately 1 p/p_0_. However, for the samples with an amine content of 50 wt.% and 70 wt.%, the hysteresis loop begins at a relative pressure of approximately 0.65 p/p_0_ and ends at a relative pressure of approximately 1 p/p_0_. This proves that, in samples with higher amine content (50 wt.% and 70 wt.%), the mesopores have a larger diameter than the mesopores in samples with lower amine content (10 wt.% and 20 wt.%); this was also true in the reference sample (TTIP_R). 

In the case of the materials heated in the high-temperature furnace (Figure 5b), and exclusively in the case of materials with a higher content of amine (50 wt.% and 70 wt.%), type IVa isotherms were obtained according to the IUPAC system and hysteresis loops of the H2b type were observed. In contrast to this, for the samples modified with 10 wt.% and 20 wt.% of DEA obtained using a tubular furnace, type II isotherms were obtained, which are characteristic of macroporous materials. Hysteresis loops of the H4 type have also been observed [41,42], what indicates the presence of pores with the shape of narrow slits [41,42].

For triethylamine-modified titanium dioxide heated in the microwave reactor, the same as in the case of diethylamine-modified titanium dioxide, type II isotherms were obtained, and hysteresis loops of the H3 type were observed (Figure 6a). For the materials modified with an amine content of 10 wt.% and 20 wt.% hysteresis loop starts at a relative pressure of 0.4 p/p_0_ and ends at a relative pressure of approximately 1 p/p_0_. Whereas for samples with an amine content of 50 wt.% and 70 wt.%, H2b type isotherms were noticed with the hysteresis loops starting at a relative pressure of approximately 0.5 p/p_0_ and ending at a relative pressure of approximately 1 p/p_0_. It means that the samples with higher amine content (50 wt.% and 70 wt.%) possessed mesopores with larger diameters than the mesopores in samples with lower amine content (10 wt.% and 20 wt.%). 

The same type of isotherm and hysteresis loop was noticed for the sample heated in the tubular furnace (Figure 6b), but only modified with amine content 50 wt.% and 70 wt.%. For the materials with lower amine content (10 wt.% and 20 wt.%) type II isotherms were obtained according to the IUPAC classification, which is characteristic of macroporous materials. Hysteresis loops of the H4 type have also been observed [41,42], which indicates the presence of pores in the shape of narrow slits [41,42].

Based on the adsorption isotherms, the textural parameters of the tested materials were calculated and presented in Table 1. Generally, all the samples heated in the microwave reactor characterized higher values of surface area and total pore volume in comparison with the materials heated in the tubular furnace. The common feature of all the samples was that they did not have micropores. 

In the case of ethylenediamine-modified titanium dioxide prepared using a microwave reactor, it was found that the content of amine in the samples had no influence on the values of specific surface areas, which ranged from 96 m^2^/g for TTIP_EDA_10%_R to 100 m^2^/g for TTIP_EDA_70%_R. Also, the values of S_BET_ did not differ significantly from the S_BET_ value of the reference sample (TTIP_R, 98 m^2^/g). In the case of diethylamine- and triethylamine-modified titanium dioxide, higher values of surface area were achieved in comparison with the EDA-modified materials. The fact that the BET-specific surface areas increased might be due to the decrease in the crystallite size of titanium dioxide (Table 1). In both cases, there was no significant effect of DEA and TEA content on S_BET_ values. For diethylamine-modified titanium dioxide, the S_BET_ values equalled around 140 m^2^/g, and for triethylamine-modified titanium dioxide, they were within the range of 140–150 m^2^/g. Slightly higher values were recorded exclusively for the cases of samples modified with 10% of amine, namely TTIP_DEA_10_%_R and TTIP_TEA_10%_R, at 160 m^2^/g and 185 m^2^/g, respectively. 

Ethylenediamine-modified titanium dioxide samples heated in the tubular furnace showed similar surface areas regardless of the amount of amine introduced. The values of S_BET_ ranged from 8 m^2^/g for TTIP_EDA_20_%_F to 10 m^2^/g for TTIP_EDA_50_%_R and were close to the values obtained for the reference material (16 m^2^/g). In the case of diethylamine- and triethylamine-modified titanium dioxide, similar to the reference material, values of S_BET_ were noticed only for the materials with the addition of 10% and 20% of amine. For materials modified with 50% and 70% of diethylamine, S_BET_ equalled 32 m^2^/g and 68 m^2^/g, whereas for materials modified with 50% and 70% of triethylamine, the S_BET_ equalled 36 m^2^/g and 48 m^2^/g, respectively. 

In general, all of the obtained materials were meso/macroporous. The total pore volume for the reference material obtained in a microwave reactor reached 0.28 cm^3^/g. Regardless of the amine used and the content of amine, the data of the total pore volume for amine-modified materials were scattered and ranged from 0.21 cm^3^/g for TTIP_DEA_50%_R to 0.40 cm^3^/g for TTIP_EDA_50%. In the case of materials obtained using a tubular furnace, the values of the total pore volume were significantly lower and the total pore volume for the reference material TTIP_F equalled 0.05 cm^3^/g. The values for the EDA, DEA and TEA-modified materials were similar. 

Table 1 presents calculated band gap energy values for titania samples modified with selected amines. It can be clearly stated that, for all modified samples fabricated with the use of a microwave reactor, slight changes in band gap energy values, and thus insignificant red-shifts of the absorption edges, are observed. Additionally, the microwave treatment did not cause the colour change, and all samples stayed white or pale beige. The white colour and the slight batochromic shift of the adsorption edge for synthesized samples under the experimental conditions presented can indicate that the microwave-assisted pressure method is insufficient for carrying out the doping of TiO_2_. However, all samples show high UV absorption due to the white colour, making them suitable for CO_2_ photoreduction with simultaneous hydrogen formation (from photocatalytic water splitting) using an excitation source with a maximum wavelength below 380 nm.

When thermal annealing at 400 °C of the samples was applied, all samples showed red-shifts in the absorption edges concerning the reference sample (TTIP_F), a consequence of narrowing the band gaps, thus decreasing E_g_ values, which proves the occurrence of TiO_2_ doping with nitrogen and/or carbon atoms. Regardless of the type of amine used, all samples showed high absorption of visible radiation due to their dark colour (from dark beige and grey to dark grey). Spadavecchia et al. [43] and Diker et al. [44] also stated that amines can be a source of nitrogen or carbon atoms, and the non-metal elements remaining after annealing in TiO_2_ can act as dopants and shift the absorption edge into the visible part of the light. From Table 1, it can be seen that the band gap energy values decrease as the amines’ theoretical concentration increases. For this preparation method, the dark colours of the synthesized samples are related to the boiling points of the amines used for modification (bp_DEA_ = 55 °C and bp_TEA_ = 89 °C). Even when the furnace is slowly heated to the required temperature (400 °C), DEA and TEA start boiling quickly and their vapours are removed with argon flow. This is also true for EDA, except that the boiling point for this amine is 117 °C, so the EDA evaporates more slowly, having a longer contact time with TiO_2_.

All the tested materials were used as photocatalysts in the photocatalytic reduction of the carbon dioxide process. Hydrogen, methane and carbon monoxide were detected in the gas phase. Although hydrogen is not a direct product of CO_2_ photoreduction, but rather a result of the water-splitting process, it is a valuable product and an essential component that is necessary for CO_2_ conversion. The content of individual gasses was expressed in μmol/g_material_/dm^3^.

**Figure 7:**
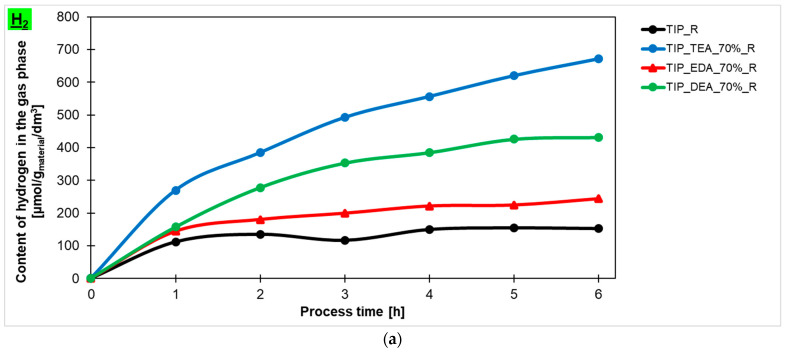

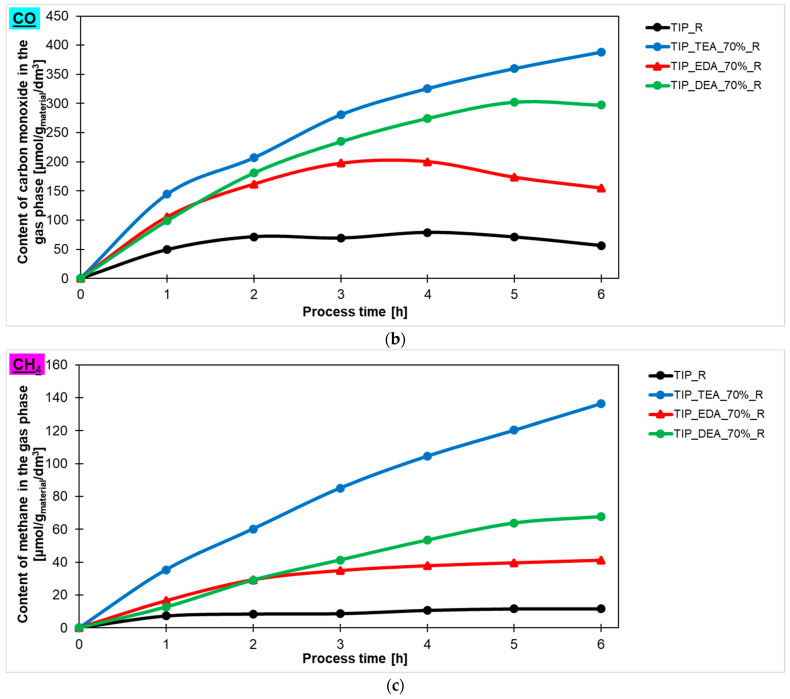
The content of hydrogen (**a**), carbon monoxide (**b**), and methane (**c**) in the gas phase obtained in the photocatalytic reduction of the carbon dioxide process using ethylenediamine-, diethylamine- and triethylamine-modified titanium dioxide heated in the microwave reactor.

The hydrogen, carbon monoxide and methane contents in the gas phase during the 6 h process, obtained using ethylenediamine-, diethylamine-, and triethylamine-modified titanium dioxide heated in the microwave reactor, are presented in Figure 7a, Figure 7b, Figure 7c, respectively. Additionally, the hydrogen, carbon monoxide and methane contents in the gas phase for the reference material prepared with ammonia water (TTIP_R) have been marked in the same Figures. It is clearly visible that the photoactivity of the studied materials depended on the type of amine used for modification. The highest photoactivity in the photoreduction of carbon dioxide process showed TEA-modified titania, and the highest content of hydrogen (671 μmol/g_material_/dm^3^), carbon monoxide (388 mol/g_material_/dm^3^) and methane (136 μmol/g_material_/dm^3^) was obtained in the gas phase using this photocatalyst. A lower content of appropriate products in the gas phase was noticed for ethylenediamine- and diethylamine-modified titanium dioxide. For ethylenediamine-modified titanium dioxide, 243 μmol/g_material_/dm^3^ of hydrogen, 154 μmol/g_material_/dm^3^ of CO and 41 μmol/g_material_/dm^3^ of CH_4_ was achieved, while, for diethylamine-modified titanium dioxide, 431 μmol/g_material_/dm^3^ of hydrogen, 297 μmol/g_material_/dm^3^ of CO and 68 μmol/g_material_/dm^3^ of CH_4_ was obtained. Based on these results, the sample modified with triethylamine has been selected for further studies. 

**Figure 8:**
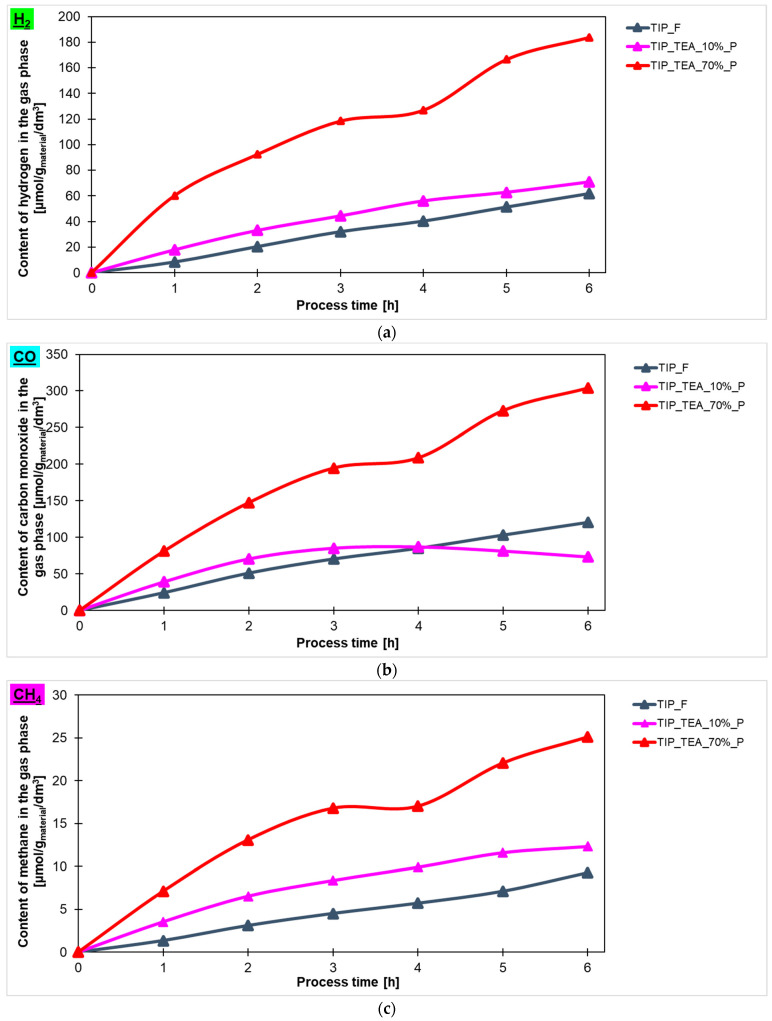
The content of (**a**) hydrogen, (**b**) carbon monoxide and (**c**) methane in the gas phase obtained in the photocatalytic reduction of carbon dioxide process using triethylamine-modified titanium dioxide heated in the microwave reactor and high-temperature furnace.

In Figure 8, the comparison between the content of hydrogen (Figure 8a), carbon monoxide (Figure 8b) and methane (Figure 8c) in the gas phase is shown and the values obtained using titanium dioxide modified with different contents of triethylamine prepared in the high-temperature furnace are presented. It is clear that higher photoactivity for hydrogen production was characterized for the samples with an amine content of 70% (183 μmol/g_material_/dm^3^). The remaining tested materials showed photoactivity at a similar level, what means that the hydrogen content in the gas phase for the sample modified with an amine content of 10% was practically the same as for the reference material. It should be noted that, in the case of carbon monoxide and methane, similar to what has been reported previously, the best results were achieved when titanium dioxide modified with an amine content of 70% was used. Generally, in the gas phase, the lowest values of the product were detected for methane. For the most photoactive material, triethylamine-modified titanium dioxide, 25 μmol/g_material_/dm^3^ of CH_4_ was obtained. 

**Figure 9:**
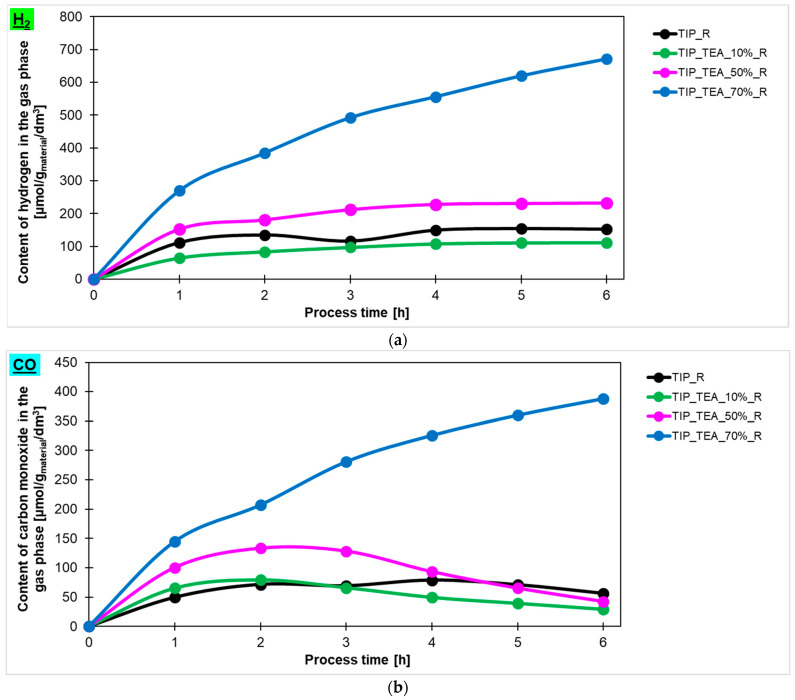

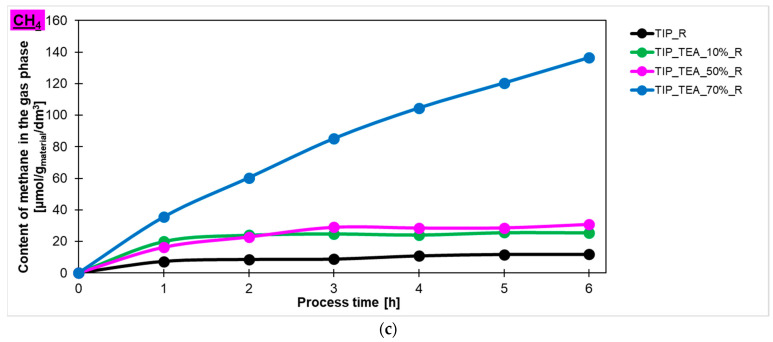
The content of (**a**) hydrogen, (**b**) carbon monoxide, (**c**) methane in the gas phase obtained in the photocatalytic reduction in the carbon dioxide process using triethylamine-modified titanium dioxide with different contents of amine and heated in the microwave reactor.

Taking into account the results described above, the influence of amine content in triethylamine-modified materials prepared in the microwave reactor on the amount of hydrogen, carbon monoxide and methane (Figure 9a, Figure 9b, Figure 9c, respectively) produced in the photocatalytic reduction of carbon dioxide process has been checked. It was found that the highest content of hydrogen in the gas phase was detected for the sample modified with 70% of amine and the amount equalled 671 μmol/g_material_/dm^3^. Lower values were noticed for the sample modified with 50% and 10% of amine—231 μmol/g_material_/dm^3^ and 111 μmol/g_material_/dm^3^, respectively. It should also be noted that the sample TTIP_TEA_10%_R exhibited lower photoactivity than the reference material. In the case of carbon monoxide and methane production, a similar dependence was observed. What is important to note is that CO production decreased for the TTIP_TEA_10%_R and TTIP_TEA_50%_R in regard to what can be related to the occupation of active sites by products or unreacted CO_2_, which prevents the reaction from proceeding further. Hydrogen production only increased in the sample modified with 70% of triethylamine (TTIP_TEA_70%_R), which suggests that saturation of the active sites in this photocatalyst occurs more slowly than for the other samples.

As is known from the available literature [45,46], photocatalytic activity depends on many factors, especially in regard to phase composition, particle size and the values of surface area. In our case, the average crystallite size and the BET-specific surface areas depended mainly on the heat treatment temperature, hence a lower content of products in the gas phase was noted for samples obtained in the high-temperature furnace. It was also visible that the use of ethylenediamine, instead of diethylamine and triethylamine, to modify titanium dioxide resulted in the formation of larger anatase crystallites for materials obtained under the same experimental conditions. This was probably due to the basicity of the individual amines and the amount of hydroxyl groups available for hydrolysis. More hydroxyl groups leads to better crystallization of TiO_2_ and greater crystallite sizes, which was observed in the case of the samples modified using EDA. This is consistent with the literature data, which states that the crystallite size depends on many parameters, including temperature, process and precursors [47,48].

## 3. Experimental Part 

### 3.1. Preparation of the Reference Material 

To prepare the titanium dioxide, 50 mL of distilled water was added dropwise to the glass beaker containing 20 mL of titanium(IV) isopropoxide (TTIP) and 5 mL of ethyl alcohol. In order to change the pH, ammonia water (25% NH_3_∙H_2_O) was dropped until the pH value was equal to 10. The whole mixture was continuously stirred for 24 h and then was left to age for 24 h. Then, the suspension was placed into a Teflon vessel and transferred to a microwave-assisted solvothermal reactor (ERTEC MAGNUM II, Wrocław, Poland). The process was conducted for 15 min under a pressure of 40 bar. Finally, the material was dried in a forced-air dryer at 80 °C, washed with distilled water, dried in a forced-air dryer at 80 °C and finally grounded in an agate mortar to a uniform consistency. The obtained sample was denoted as TTIP_R. Simultaneously, the sample was also taken without microwave treatment. The procedure was similar, with the exception that, after the ageing step, the material was dried in a forced-air dryer at 80 °C, grounded in an agate mortar and subjected to the heat treatment, which was performed in a high-temperature furnace (HST 12/400 Carbolite, Derbyshire, UK). For this purpose, the obtained powder was transferred into a quartz boat, placed in a high-temperature furnace and heated under an argon atmosphere at a temperature rising from 20 to 400 °C with a heating rate of 10 °C/min. When a temperature of 400 °C was reached, the heating process was continued for 1 h. Afterwards, the sample was cooled to room temperature under an argon atmosphere. The final product was washed with distilled water, dried in a forced-air dryer at 80 °C and finally grounded in an agate mortar to a uniform consistency. The obtained sample was denoted as TTIP_F.

### 3.2. Modification of Titanium Dioxide with Amines 

To prepare amine-modified titanium dioxide, 50 mL of distilled water was added dropwise to the glass beaker containing 20 mL of titanium(IV) isopropoxide (TTIP) and 5 mL of ethyl alcohol. Then, the appropriate amine (ethylenediamine, diethylamine and triethylamine), in an amount of 10 wt.%, 20 wt.%, 50 wt.% and 70 wt.%, was added. The whole mixture was continuously stirred for 24 h, and was then left to age for 24 h. Then, the sample was placed into a Teflon vessel and transferred to a microwave reactor (ERTEC MAGNUM II). The process was conducted for 15 min under a pressure of 40 bar. Finally, the material was dried in a forced-air dryer at 80 °C, washed with distilled water, again dried in a forced-air dryer at 80 °C and finally grounded in an agate mortar to a uniform consistency. The obtained samples were denoted as TTIP_EDA_10%_R, TTIP_EDA_20%_R, TTIP_EDA_50%_R, TTIP_EDA_70%_R (ethylenediamine-modified titanium dioxide); TTIP_DEA_10%_R, TTIP_DEA_20%_R, TTIP_DEA_50%_R, TTIP_DEA_70%_R (diethylamine-modified titanium dioxide); and TTIP_TEA_10%_R, TTIP_TEA_20%_R, TTIP_TEA_50%_R, TTIP_TEA_70%_R (triethylamine-modified titanium dioxide). 

Simultaneously, samples without microwave treatment were also made. The procedure was similar, with the exception that, after the ageing step, the material was dried in a forced-air dryer at 80 °C, grounded in an agate mortar and subjected to the heat treatment, which was performed in a high-temperature furnace (HST 12/400 Carbolite). For this purpose, the obtained powder was transferred into a quartz boat, placed in a high-temperature furnace and heated under an argon atmosphere, with the temperature rising from 20 to 400 °C at a heating rate of 10 °C/min. When a temperature of 400 °C was reached, the heating process was continued for 1 h. Afterwards, the sample was cooled to room temperature under an argon atmosphere. The final product was washed with distilled water, dried in a forced-air dryer at 80 °C and finally grounded in an agate mortar to a uniform consistency. The obtained samples were denoted as TTIP_EDA_10%_F, TTIP_EDA_20%_F, TTIP_EDA_50%_F, TTIP_EDA_70%_F (ethylenediamine-modified titanium dioxide); TTIP_DEA_10%_F, TTIP_DEA_20%_F, TTIP_DEA_50%_F, TTIP_DEA_70%_F (diethylamine-modified titanium dioxide); TTIP_TEA_10%_F, TTIP_TEA_20%_F and TTIP_TEA_50%_F, TTIP_TEA_70%_F (triethylamine-modified titanium dioxide). 

### 3.3. Photocatalytic Reduction of Carbon Dioxide

The processes were conducted in a gas phase bottle-shaped reactor made of glass. The working volume of the reactor was 766 cm^3^. A 150 W medium-pressure mercury lamp TQ150 Z3 (Heraeus, Hanau, Germany) was used in the photocatalytic tests. It was characterized by the wide range of both UV and visible light in the range of 250–600 nm, with the maximum at 365 nm. The lamp was placed in a quartz condenser. It was constantly cooled with water by a chiller equipped with a pump with a controlled temperature of 18 °C (Minichiller 280 OLÉ, Huber, Offenburg, Germany). The reactor was placed in a thermostatic chamber to maintain a stable temperature (20 °C) and exclude light sources. A measurement of 10 cm^3^ of distilled water and glass fibre with the tested photocatalyst were placed in the reactor. The reactor was purified with pure CO_2_ (Messer, Police, Poland) for 16 h to eliminate the air. During the whole process, the gas in the reactor was constantly mixed with the pump (flow rate of 1.6 dm^3^/h). The process was performed at 20 °C and tested for 6 h. The gas samples for analysis were collected every 1 h. The gas phase composition was analyzed using an SRI 310C gas chromatograph (SRI Instruments, Torrance, CA, USA) equipped with a column with a molecular sieve with a mesh size of 5Ä and a HID detector (Helium Ionization Detector). The carrier gas was helium. The analyses were performed under isothermal conditions at a temperature of 60 °C. The gas flow through the column was 60 cm^3^/min, and the volume of the test gas was 1 cm^3^. The content of the component in the gas phase was calculated in successive measurements based on the calibration curve. 

### 3.4. Characterization Methods

The phase composition of the prepared samples was determined using the X-ray diffraction method Cu Kα radiation (λCu Kα = 0.1540 nm) on an Empyrean (Panalytical, Malvern, UK). The identification of the crystalline phases was performed using HighScore+ and the ICDD PDF-4+ 2015 database. The average crystallite size was calculated according to the following Scherrer equation based on obtained X-ray powder diffraction patterns:

D = (k∙λ)/(β·cosθ)

where D—the average crystallite size in the direction perpendicular to the (hkl) reflection plane; k—a constant close to unity, dependent on the shape of the crystallite; λ—the X-ray wavelength; β—the peak broadening; θ—the XRD peak position.

To perform the low-temperature nitrogen adsorption/desorption studies, the equipment QUADRASORB evoTM gas sorption automatic system (Quantachrome Instruments, Anton Paar, Austria) was used together with a MasterPrep multi-zone flow/vacuum outgassing system under a vacuum of 1 × 10^−5^ mbar from Quantachrome Instruments (Boynton Beach, FL, USA). On the basis of the obtained adsorption/desorption isotherms, the specific surface area (S_BET_) and pore volumes of the obtained materials were determined. Prior to analysis, 150 mg of the material was weighed and pre-dried at 90 °C in a laboratory dryer. The dried samples were transferred into measuring cells and degassed using MasterPrep (Ouantachrome Instruments, USA) at 100 °C for 12 h. The Brunauer–Emmett–Teller (BET) equation was used to determine the surface areas (S_BET_), which were determined in the relative pressure range of 0.05–0.2. The total pore volume (V_total_) was calculated from the volume of nitrogen held at the highest relative pressure (p/p_0_ = 0.99). The volume of micropores (V_micro_ < 2 nm) with dimensions smaller than 2 nm was calculated as a result of integrating the pore volume distribution function using the DFT method; a mesopore volume (V_meso_) with dimensions from 2 to 50 nm was calculated from the difference in the total pore volume (V_total_) and the volume of micropores (V_micro_ < 2 nm).

The *band gap* of the reference and all amine-modified titania materials was determined from the optical *absorption* spectra by means of a Jasco V-650 spectrometer (JASCO International Co., Tokyo, Japan) equipped with a PIV-756 integrating sphere accessory spectrometer (JASCO International Co., Tokyo, Japan) for diffuse reflectance measurements. Barium sulphate (BaSO_4_ pure p.a., Avantor Performance Materials Poland S.A., Gliwice, Poland) was used as a reference for determining the baseline. The Tauc plot was used to estimate the value of the semiconductor band gap energy.

## 4. Conclusions

Modification of titanium dioxide using ethylenediamine, diethylamine, and triethylamine heated in the microwave reactor or high-temperature furnace has been studied. Based on the X-ray diffraction method, it was found that, in all the tested materials, only the anatase phase was identified. The average crystallite size was higher for the raw material and materials modified with amines and heated in the high-temperature furnace instead of microwave reactor, due to the higher processing temperature. Regardless of the heating method, decrease of the average crystallite size after modification with amines in comparison with raw materials was noticed, apart from ethylenediamine-modified titanium dioxide heated in the microwave reactor, for which slightly higher values were noticed. Generally, all the samples heated in the microwave reactor were characterized with higher values of surface area, total pore volume and band gap energy in comparison with the materials heated in the tubular furnace. Modifying samples with amines utilizing microwave-assisted pressure treatment did not alter the E_g_ values, as can be indirectly evidenced by the white colour of the samples, making them capable of absorbing the UV radiation. On the other hand, thermal amine modification of titania at 400 °C using the high-temperature furnace leads to colour changes and the red-shift of the adsorption edge, which indicates the doping of TiO_2_ with carbon and/or nitrogen. The main product detected in the gas phase was hydrogen, and the highest content was achieved using triethylamine-modified titanium dioxide with 70% of amine content and heated using microwaves. 

## Figures and Tables

**Table 1:** The average crystallite size calculated according to the Scherrer equation, textural properties of the amine-modified TiO_2_ calculated based on low-temperature nitrogen adsorption isotherms and band gap energy estimated from Tauc’s plots.

		Amine Content	Average Crystallite Size	S_BET_	TPV	V_micro_	V_meso_	Eg
		[wt.%]	[nm]	[m^2^/g]	[cm^3^/g]	[cm^3^/g]	[cm^3^/g]	[eV]
Microwave reactor								
TTIP_R		0	14.3	98	0.28	0.01	0.27	3.22
TTIP_EDA_10%_R		10	15.4	96	0.28	0.00	0.28	3.20
TTIP_EDA_20%_R		20	16.6	93	0.30	0.00	0.30	3.17
TTIP_EDA_50%_R		50	16.0	86	0.40	0.00	0.40	3.22
TTIP_EDA_70%_R		70	16.9	100	0.34	0.01	0.33	3.20
TTIP_DEA_10%_R		10	8.5	160	0.27	0.01	0.26	3.23
TTIP_DEA_20%_R		20	9.7	146	0.25	0.01	0.24	3.20
TTIP_DEA_50%_R		50	10.2	152	0.23	0.00	0.23	3.15
TTIP_DEA_70%_R		70	9.3	148	0.25	0.01	0.24	3.18
TTIP_TEA_10%_R		10	7.1	185	0.33	0.01	0.32	3.26
TTIP_TEA_20%_R		20	10.2	143	0.22	0.01	0.21	3.16
TTIP_TEA_50%_R		50	9.1	152	0.27	0.01	0.26	3.23
TTIP_TEA_70%_R		70	9.4	138	0.25	0.01	0.24	3.23
High-temperature furnace								
TTIP_F		0	21.9	16	0.05	0.00	0.05	3.25
TTIP_EDA_10%_F		10	13.8	10	0.06	0.00	0.06	2.54
TTIP_EDA_20%_F		20	11.6	8	0.04	0.00	0.04	2.45
TTIP_EDA_50%_F		50	10.6	12	0.02	0.00	0.02	2.37
TTIP_EDA_70%_F		70	9.0	9	0.04	0.00	0.04	2.26
TTIP_DEA_10%_F		10	12.7	15	0.12	0.00	0.12	2.92
TTIP_DEA_20%_F		20	15.1	9	0.03	0.00	0.03	2.92
TTIP_DEA_50%_F		50	17.0	32	0.03	0.01	0.02	2.33
TTIP_DEA_70%_F		70	14.0	68	0.06	0.01	0.05	2.59
TTIP_TEA_10%_F		10	10.3	19	0.06	0.00	0.06	2.75
TTIP_TEA_20%_F		20	14.5	6	0.04	0.00	0.04	2.74
TTIP_TEA_50%_F		50	14.3	36	0.03	0.01	0.02	2.56
TTIP_TEA_70%_F		70	13.8	48	0.05	0.01	0.04	2.32

## Data Availability

No new data were created or analyzed in this study. Data sharing is not applicable to this article.
